# Large ethnic variations in recommended physical activity according to activity domains in amsterdam, the netherlands

**DOI:** 10.1186/1479-5868-7-85

**Published:** 2010-11-29

**Authors:** Jeroen SL de Munter, Irene GM van Valkengoed, Charles Agyemang, Anton E Kunst, Karien Stronks

**Affiliations:** 1 Academic Medical Center, Dept. of Public Health, University of Amsterdam, Amsterdam

## Abstract

**Purpose:**

The level of recommended physical activity (PA) is met less frequently by people from some ethnic minorities than others. We explored whether these differences in recommended PA between ethnic minority groups and the general population varied by domain and type of culturally-specific activity.

**Methods:**

Participants were sampled from the population based SUNSET study and were from ethnic Dutch (n = 567), Hindustani-Surinamese (n = 370) and African-Surinamese (n = 689) descent. The validated SQUASH-questionnaire measured PA for the following domains: commuting, occupation, household, leisure time. Culturally-specific activities were added as extra question within the leisure time domain. The effect of each domain on ethnic differences in recommended PA prevalence was examined by odds-ratio (OR) analysis through recalculating recommended PA, while, in turn, excluding the contribution of each domain.

**Results:**

In the ethnic Dutch population, more vigorous PA in commuting and leisure time was reported compared to the Surinamese groups. The Hindustani-Surinamese and African-Surinamese reported more walking as commuting activity, while the Dutch group reported cycling more frequently. Ethnic differences in recommended PA became smaller in both Surinamese groups compared with the Dutch after removing commuting activity, for example, in Hindustani-Surinamese men (OR = 0.92, 95%CI: 0.62-1.37 vs. OR = 1.33, 0.89-2.00) and women (OR = 1.61, 1.12-2.32 vs. OR = 2.03, 1.41-2.92). Removing occupational activity resulted in larger ethnic differences in both groups compared with the Dutch. Smaller effects were found for yoga and dancing, leisure time and household activities.

**Conclusion:**

This study shows that differences in PA between ethnic minority groups and the general population vary according to the activity domain. The results indicate that including all relevant domains and activities is essential for assessment of ethnic differences in recommended PA.

## Introduction

Physical activity (PA) levels are known to differ between ethnic groups. Several studies from West European and North American countries show that people from ethnic minorities are less active than the general population [1-3]. For example, African Americans report higher levels of physical *in*activity during leisure time compared to their White-American counterparts in a similar social class [4]. Moreover, South Asians living in the UK report lower levels of PA and are less likely to meet the PA guideline compared with the general population [5]. In contrast, African Caribbeans living in the UK showed similar levels of adherence to the PA recommendation but reported lower levels of brisk walking compared with the general population [6].

There is evidence that shows that the level of PA in people from ethnic minorities varies between domains, e.g. higher levels of PA have been reported in some of these groups for the domain of occupational PA compared with the general population [7,8]. In contrast, the gap in PA seems to be relatively large for leisure time PA which puts some ethnic minority groups at a disadvantage [9]. These lower levels of leisure time physical activity in ethnic minority compared to general population are not only explained by differences in socioeconomic position [1]. Differences may also be related to socio-cultural factors, such as preferences for specific types of activities [10,11]. This raises the question of how this variation in ethnic differences across domains relates to the total level of PA in a particular group. If, for example, the level of activity among some ethnic minority populations is higher in the occupational domain, and relatively low in leisure time, what does this mean for the level of recommended PA (the level at which we see a beneficial effect on health)? And, vice versa, if ethnic minorities score lower on the recommended level of PA, whereas the amount of occupational PA is relatively high, how large are the ethnic differences in PA outside of the occupational domain? So far, the pattern underlying the ethnic differences in recommended PA, i.e. the specific differences between activity domains and the activities within those domains that influence the level of recommended PA, have not been studied extensively. Therefore, the aim of this study is to identify how much each activity domain contributes to the recommended level of PA and to observe the extent to which these domains influence the ethnic differences in recommended PA.

Using data from the Dutch SUNSET study on Hindustani-Surinamese, African-Surinamese and ethnic Dutch populations, we first describe ethnic differences in the various domains of PA (commuting, occupational, household and leisure time) and the frequency, intensity, and duration of activities within each domain. Subsequently, we calculate the prevalence of recommended PA and the ethnic differences between the population groups. Finally, we assess how the specific domains and the culturally-specific activities have contributed to the ethnic differences found in recommended PA.

## Methods

### Study population & data collection

We used data from the population based SUNSET (Surinamese in the Netherlands: Study on health and Ethnicity) study, which was set up to gain insight into the cardiovascular risk profile in people aged 35-60 years in three ethnic groups (Hindustani-Surinamese, African-Surinamese and Dutch). The recruitment and design of the study have been described elsewhere [12,13]. In brief, potential participants were randomly sampled (n = 2,975) from the population register of Amsterdam, the Netherlands. Between 2001 and 2003, potential participants - matched by sex and presumed ethnicity - were approached at home for a structured face-to-face interview with a trained interviewer. The interview contained questions on lifestyle, migration history, demographic variables, and general health status. After the interview, participants of Hindustani-Surinamese, African Surinamese and Dutch descent were invited for a physical examination at a local health center. The response to the interview was 60% in the Surinamese and 61% in the Dutch populations. Resulting in a total of n = 1,626 eligible participants from Dutch (n = 567), Hindustani-Surinamese (n = 370) and African-Surinamese (n = 689) descent that were included in this study. The Dutch population was older than the Surinamese; the mean age of the Dutch participants was 47.6 years, while the mean age of the Hindustani-Surinamese and African-Surinamese was 44.5 and 43.6 years, respectively. The study was approved by the Institutional Review Board of the Academic Medical Centre of the University of Amsterdam. All participants provided a written informed consent.

#### Migration history & ethnicity

Many people from the Surinamese population migrated to the Netherlands in 1975, when Suriname gained its independence. The official language of the republic of Suriname is Dutch. Approximately 80% of the Surinamese population living in the Netherlands is either from African or South Asian/Hindustani descent. In this study, the populations are called African-Surinamese and Hindustani-Surinamese, respectively.

For the sampling procedure, those people born in the Netherlands and whose parents were both born in the Netherlands were presumed to be of Dutch origin. Those people whose parents were both born in Suriname and those who were born in Suriname and who had at least one parent who was born in Suriname were considered to be of Surinamese origin. Subsequently, ethnicity was determined during the interview according to self-identified ethnicity (Hindustani-Surinamese, African-Surinamese, Dutch or other).

In those cases where information on the self-identified ethnicity of the individual was lacking or unclear, then information on the ethnic group of the mother, the father and the mother's ancestry was used to classify participants.

#### Physical activity questionnaire

The SQUASH questionnaire was used to measure the participants' level of PA. The questionnaire covers questions similar to those in the long-format International Physical Activity Questionnaire (IPAQ) and has been validated for the Dutch population [14]. The SQUASH was not translated or simplified as the Surinamese population in the Netherlands has a good understanding of the Dutch language; less than 2% of participants reported having trouble with the Dutch language [unpublished]. In addition, most participants have lived in the Netherlands for more than 20 years and migrated directly from Suriname, where the official language is Dutch. The SQUASH questionnaire requires participants to recall their normal pattern of PA during an average week and drawn from the past few months. It covers PA for four 'domains' as follows: 1) commuting, 2) occupational or school-related, 3) household, and 4) leisure time. Participants were asked to indicate the frequency (times per week), self-reported intensity (light, moderate or vigorous) and average duration of the activity per day for each domain. In the SQUASH questionnaire the commuting PA was measured by questions about walking and/or cycling to and from work. The questions concerning occupational and household related physical activities were divided into light and vigorous intensity categories due to possible cognitive difficulties when people self-report their level of intensity for these activities [14]. As light intensity household and occupational tasks do not add much to the recommended level of PA, we show only the frequency of these tasks at vigorous intensity in the tables. For leisure time PA, participants were asked about four physical activities (walking, cycling, gardening and do-it-yourself) that are common in the Netherlands. For the purpose of this study, two extra types of leisure time activity (yoga and dancing) were added because they are commonly practiced in the study population. We also supplemented the SQUASH questionnaire with an open-ended question on the practice of other leisure time activities (no sports), to enable activities not covered by the questionnaire to be reported. Finally, in the last section, participants could report up to four leisure time sports, either organized or non-organized with accompanying frequency, intensity and duration.

### Calculating prevalence of recommended physical activity

The SQUASH questionnaire comes with a standard analysis syntax to calculate the prevalence of recommended PA according to the Dutch guideline (or "combinorm") [14]. The Dutch guideline is similar with the international guideline for recommended PA, which states that all adults should be physically active at a moderate intensity for at least 30 minutes each day for a minimum of 5 days per week or at a vigorous intensity for a minimum of 20 minutes on at least three days per week [15,16].

Reported physical activities were coded using the Compendium of Ainsworth and subsequently divided into three intensity categories based on their metabolic equivalents (MET) depending on age; for younger people (< 55: light < 4.0, moderate 4.0 to < 6.5 and vigorous ≥ 6.5) and older people (≥ 55, light < 3.0, moderate 3.0 to < 5.0 and vigorous ≥ 5.0) [17,18]. This MET category was combined with self-reported intensity for each activity, resulting in a combined intensity score ranging from 1 to 9, with 1 being light MET and light self-reported intensity to 9 being vigorous MET and vigorous self-reported intensity [14].

Finally, participants were scored as meeting the recommended level of physical activity when the combined intensity score was at least moderate (≥ 3) and the combined duration of all activities was at least 30 minutes per day on at least 5 days per week or when the combined intensity score was vigorous (≥ 6) and the combined duration of vigorous activity was at least 20 minutes on at least 3 days per week. Reported average duration of activity that was less than 10 minutes per day was not included in this calculation.

### Other variables

Education level was determined by the highest education achieved and was grouped as: secondary school and below i.e. ≤ 12 years of education (low), and vocational school and above i.e. ≥ 13 years of education (high). Educational level has proven to be the best indicator of socio-economic status in the Netherlands [19].

### Analyses

The PA characteristics of participants and the prevalence of recommended PA are presented and are age-standardized to the total Amsterdam population in 2001. Results are stratified by gender. For the presentation of leisure time sports, self-reported intensity and duration were averaged for those participants who reported more than one sport.

Chi-square was used to test differences between ethnic groups for the categorical variables (frequency and intensity). Differences in skewed continuous variables were analyzed by means of the Kruskall-Wallis rank test.

We used multivariate logistic regression models to calculate age-adjusted odds ratios (OR) with robust variance and accompanying 95% confidence intervals to examine the group differences regarding the prevalence of recommended PA.

Furthermore, the contribution of each activity domain to the level of recommended PA was assessed by recalculating the recommended level of PA. We excluded, one by one, the contribution of physical activity from one of the four domains (commuting, occupational, household, leisure time) in the calculation for recommended physical activity. This resulted in four 'variants' of recommended physical activity; each one of these variants lacking information from one specific domain. These variants of recommended physical activity were subsequently put in the multivariate model, adjusted by age. Which enabled us to determine the size of the ethnic differences in terms of ORs for recommended PA in the ethnic groups, compared to the Dutch group. By comparing the determined ORs for the variants with the OR based on the original level of recommended PA (i.e. including all domains and activities) we could assess the change in the association due to the specific domains or activities. Finally, we added education as covariate to our models to identify how much of the ethnic differences in meeting the recommended level of physical activity could be explained by differences in education. All analyses were computed with STATA 9.2 (Stata Corp. College Station, Texas). P values < 0.05 were considered statistically significant.

## Results

### 1. Domains and types of physical activity

#### Commuting

Table 1 shows the frequency, intensity and duration of reported physical activities within the domain of commuting. African-Surinamese participants reported a higher frequency of walking to work, while the Dutch reported more cycling to work. The reported intensity levels for the activity in the various ethnic groups differed regarding walking in women and cycling in men. In general, Dutch participants reported a higher prevalence of vigorous activity compared to the ethnic minority groups. Differences in duration were only found for cycling and only for men: Dutch men reported a longer duration in cycling to work compared to African-Surinamese and Hindustani-Surinamese men.

**Table 1:** Frequency^a^, intensity^a ^and duration of physical activity in *commuting*.

		Men			Women		
		Dutch n = 276	Hindustani- Surinamese n = 162	African- Surinamese n = 231	Dutch n = 291	Hindustani- Surinamese n = 208	African- Surinamese n = 458
**Walking**	frequency	**24.8**	**16.0**	37.1**	**19.7**	**35.0**	41.1**
	intensity (% moderate)	14.2	11.6	22.0	**11.8**	**28.6**	27.8**
	(% vigorous)	8.5	1.7	6.8	**7.2**	**3.8**	**10.0**
	duration (median, min/week)	100	100	90	100	100	100
**Cycling**	frequency	**32.4**	**6.3**	13.2**	**31.3**	**8.2**	17.3**
	intensity (% moderate)	**15.8**	**4.6**	9.4*	23.1	6.4	11.6
	(% vigorous)	**14.2**	**1.7**	**2.5**	6.7	1.6	4.4
	duration (median, min/week)	**150**	**60**	120*	120	100	120

^a^Age-standardized to Amsterdam population. Duration is given for both medium and vigorous intensity physical activity. Difference in duration: Kruskall-Wallis rank test for difference. Difference in frequency and intensity: Chi-square test of independence between groups. *p < 0.05. **p < 0.01.

#### Occupational and household

No differences were observed between the Dutch and the African-Surinamese and Hindustani-Surinamese groups for the frequency of vigorous occupational and household PA (table 2). However, both Surinamese groups reported a longer duration of vigorous occupational PA compared to the Dutch group.

**Table 2:** Intensity^a ^and duration of physical activity in *occupational *and *household *physical activity

		Men			Women		
		Dutch	Hindustani- Surinamese	African- Surinamese	Dutch	Hindustani- Surinamese	African- Surinamese
**Occupational**	intensity (% vigorous)	32.2	39.1	34.2	23.7	24.9	26.7
	duration (median, hours/week)	**20**	**36**	36*	**18**	**30**	28.5*
							
**Household**	intensity (% vigorous)	48.4	59.9	55.8	58.6	59.3	60.9
	duration (median, hours/week)	**2**	**2**	2*	3	3	3

^a^Age-standardized to Amsterdam population. Difference in intensity: Chi-square test of independence between groups. Difference in duration: Kruskal-Wallis rank test for difference. *p < 0.05.

#### Leisure time

In general, the reported frequency of leisure time PA was higher in the Dutch participants compared to the Surinamese groups (table 3). Yoga, however, was more frequently reported by Hindustani-Surinamese men than other men and dancing was more frequently reported by African-Surinamese men and women than in the other groups. The proportion of vigorous PA was highest in Dutch men and women for sports, walking, cycling and dancing. Regarding the duration of activities, African-Surinamese women reported longer duration for leisure time sport and dancing compared with Dutch women.

**Table 3:** Frequency^a^, intensity^a ^and duration of physical activity in *leisure time *physical activity.

		Men			Women		
	Intensity, %	Dutch	Hindustani- Surinamese	African- Surinamese	Dutch	Hindustani- Surinamese	African- Surinamese
**Sports**	frequency	**41.3**	**28.9**	36.3*	**45.1**	**25.1**	25.6**
	intensity (% moderate	**11.5**	**19.5**	24.2**	**20.1**	**19.1**	18.1**
	(% vigorous)	**26.2**	**4.9**	**7.8**	**19.6**	**1.6**	**6.2**
	duration (median, min/week)	180	180	240	**137.5**	**120**	180*
**Walking**	frequency	54.8	60.2	57.0	64.4	69.1	63.3
	intensity (% moderate)	**33.0**	**46.4**	40.5**	42.5	58.1	38.7
	(% vigorous)	**8.8**	**3.2**	**3.9**	8.7	4.3	6.3
	duration (median, min/week)	180	210	180	210	240	180
**Cycling**	frequency	**48.8**	**28.7**	38.8**	**51.8**	**23.3**	30.5**
	intensity (% moderate)	**30.6**	**24.3**	26.4**	42.7	19.3	24.3
	(% vigorous)	**14.4**	**1.5**	**5.2**	7.5	2.6	2.5
	duration (median, min/week)	120	135	150	120	120	120
**Gardening**	frequency	**18.1**	**9.3**	13.4*	**26.7**	**11.8**	11.2**
	intensity (% moderate)	8.7	7.1	8.4	15.8	8.1	5.6
	(% vigorous)	4.4	0	1.3	4.0	0.4	0.5
	duration (median, min/week)	60	60	60	60	105	60
**DIY**	frequency	**37.2**	**22.5**	26.4*	**20.3**	**10.2**	8.9**
	intensity (% moderate)	28.8	13.4	19.6	14.1	5.7	6.0
	(% vigorous)	2.2	1.0	0	2.5	1.3	0.2
	duration (median, min/week)	180	120	90	120	60	60
**Yoga**	frequency	**3.3**	**9.1**	3.9*	4.0	4.7	4.3
	intensity (% moderate)	2.0	8.0	2.8	1.7	4.2	2.7
	(% vigorous)	0.5	0.6	0.3	1.2	0	0.9
	duration (median, min/week)	75	100	180	90	120	90
**Dancing**	frequency	**11.9**	**7.0**	27.6**	**15.3**	**12.2**	29.4**
	intensity (% moderate)	**6.9**	**5.9**	20.2**	**7.7**	**9.7**	20.0**
	(% vigorous)	**4.7**	**1.2**	**2.6**	**7.4**	**1.6**	**6.0**
	duration (median, min/week)	120	120	120	**82.5**	**120**	120*

^a^Age-standardized to Amsterdam population. Duration is given for both medium and vigorous intensity physical activity. Difference in duration: Kruskall-Wallis rank test for difference. Difference in frequency and intensity: Chi-square test of independence between any vs. none and medium vs. vigorous groups. *p < 0.05. **p < 0.01. DIY: Do-it-yourself

### 2. Prevalence of recommended physical activity

We observed a lower prevalence of recommended PA in Hindustani-Surinamese women (42.9%) compared to Dutch women (61.7%, table 4). In African-Surinamese women, the prevalence of recommended PA was 55.2%. A similar pattern was observed in men, i.e. the estimates were lower in Hindustani-Surinamese (58.2%) and African-Surinamese (58.0%) compared to Dutch men (66.2%). The ethnic difference in terms of odds ratios for *not *meeting the PA recommendation, compared to Dutch participants, was 1.33 (0.89-2.00) in Hindustani-Surinamese men and 2.03 (1.41-2.92) in Hindustani-Surinamese women. In African-Surinamese men, the odds ratio was 1.43 (0.99-2.07) and in African-Surinamese women 1.28 (0.94-1.74) compared with Dutch participants.

**Table 4:** Ethnic differences in the prevalence and association (odds ratio) for the recommended level of physical activity between the Surinamese and the Dutch population, before and after excluding physical activity from specific domains or culturally-specific types of physical activity.

	Prevalence (95% CI)^a^			Odds ratio (95% CI)^b^				
	Dutch	Hindustani- Surinamese	African- Surinamese	Dutch	Hindustani- Surinamese	African- Surinamese	Hindustani- Surinamese	African- Surinamese
**Men**					**model 1**	**model 1**	**model 2**	**model 2**
Recommended physical activity	66.2 (60.0-71.9)	58.2 (50.3-65.8)	58.0 (51.1-64.6)	1.00	1.33 (0.89-2.00)	1.43 (0.99-2.07)	1.36 (0.88-2.09)	1.46 (0.99-2.15)
								
Excl. Commuting activity	54.7 (48.3-61.0)	56.1 (48.2-63.7)	55.4 (48.4-62.1)	1.00	0.92 (0.62-1.37)	1.01 (0.71-1.45)	1.00 (0.66-1.53)	1.08 (0.74-1.58)
Excl. Occupational activity	52.8 (46.4-59.0)	33.1 (26.2-40.8)	41.3 (34.6-48.3)	1.00	**2.02 (1.34-3.04)**	**1.56 (1.09-2.25)**	**1.86 (1.20-2.88)**	**1.49 (1.02-2.17)**
Excl. Household activity	62.7 (56.3-68.6)	51.3 (43.4-59.1)	56.2 (49.3-62.9)	1.00	**1.52 (1.02-2.27)**	1.35 (0.94-1.95)	1.50 (0.98-2.30)	1.34 (0.92-1.97)
Excl. Leisure time activity	45.6 (39.3-52.0)	38.6 (31.3-46.5)	40.7 (34.1-47.6)	1.00	1.30 (0.87-1.94)	1.27 (0.88-1.82)	**1.54 (1.00-2.39)**	**1.46 (1.00-2.14)**
Excl. Yoga, dancing activity	65.5 (59.3-71.2)	54.9 (47.0-62.6)	55.0 (48.0-61.7)	1.00	1.48 (0.99-2.21)	**1.55 (1.07-2.23)**	**1.55 (1.01-2.39)**	**1.61 (1.09-2.36)**
								
**Women**								
Recommended physical activity	61.7 (55.5-67.5)	42.9 (36.2-49.8)	55.2 (50.3-60.0)	1.00	**2.03 (1.41-2.92)**	1.28 (0.94-1.74)	**1.84 (1.26-2.67)**	1.25 (0.92-1.70)
								
Excl. Commuting activity	52.2 (46.0-58.3)	40.3 (33.7-47.3)	51.4 (46.5-56.3)	1.00	**1.61 (1.12-2.32)**	1.04 (0.77-1.40)	**1.56 (1.07-2.26)**	1.04 (0.77-1.40)
Excl. Occupational activity	53.6 (47.4-59.7)	27.5 (21.8-34.0)	39.8 (35.1-44.7)	1.00	**2.82 (1.93-4.14)**	**1.77 (1.31-2.40)**	**2.60 (1.75-3.85)**	**1.71 (1.26-2.32)**
Excl. Household activity	55.2 (48.9-61.2)	39.1 (32.6-46.0)	48.6 (43.7-53.5)	1.00	**1.88 (1.30-2.70)**	1.33 (0.98-1.79)	**1.65 (1.13-2.39)**	1.28 (0.95-1.74)
Excl. Leisure time activity	41.0 (35.0-47.2)	28.0 (22.2-34.6)	34.4 (29.9-39.1)	1.00	**1.63 (1.11-2.40)**	1.23 (0.90-1.68)	1.48 (0.99-2.20)	1.20 (0.88-1.64)
Excl. Yoga, dancing activity	60.3 (54.2-66.2)	42.0 (35.3-48.9)	51.7 (46.8-56.6)	1.00	**1.94 (1.35-2.79)**	**1.38 (1.02-1.87)**	**1.74 (1.20-2.53)**	1.34 (0.99-1.82)

^a ^Prevalence is age-standardized to Amsterdam total population. ^b ^Odds ratio is determined by age-adjusted logistic regression, the odds ratio is given for not-adhering to the recommended level of physical activity. CI confidence interval. Model 1 adjusted for age. Model 2. Model 1 plus further adjustment for level of education (high vs. low)

### 3. Effect of domains and culturally-specific activities

The difference between the ethnic minority and the Dutch population regarding the prevalence of recommended PA was *smaller *after the domain of commuting was excluded (table 4). Activity in the domain of commuting contributed more towards recommended PA in the Dutch participants compared to the Surinamese. In Dutch men, commuting contributed to recommended PA prevalence by 11.5% and in Dutch women by 9.5% (see table 4), whereas in Hindustani-Surinamese men this was 2.1% and in Hindustani-Surinamese women 2.6%. In the African-Surinamese population the contribution for commuting was 2.6% in men and 3.8% in women. The odds ratio for ethnic differences in *not *meeting the recommended level of PA was smaller for the Hindustani-Surinamese men (OR 0.92, 95% CI 0.62-1.37) and women (1.61, 1.12-2.32) and for the African-Surinamese men (1.01, 0.71-1.45) and women (1.04, 0.77-1.40) after the contribution of commuting PA was removed.

In contrast, ethnic differences in the prevalence of recommended PA *increased *(compared with the Dutch group) after occupational PA for men and women in both Surinamese groups was excluded (table 4). This reflects the relatively high levels of occupational PA in the Surinamese group. The odds ratio for not meeting the PA recommendation increased from 1.33 to 2.02 in Hindustani-Surinamese men and 2.03 to 2.82 in Hindustani-Surinamese women after excluding PA in the occupational domain (table 4).

The effect of excluding yoga and dancing differed per ethnic group. Compared with the Dutch participants, this difference decreased in Hindustani-Surinamese women but increased in the other groups (table 4). The ethnic difference in recommended PA after excluding yoga and dancing was 1.55 (1.07-2.23) in African-Surinamese men and 1.38 (1.02-1.87) in women and 1.48 (0.99-2.21) for Hindustani-Surinamese men and 1.94 (1.35-2.79) for women compared to the Dutch participants (table 4).

The pattern of ethnic differences for meeting the recommended physical activity remained similar after adjustment for education (table 4 model 2).

## Discussion

### Key findings

Our study explored the ethnic variation in PA according to the domains and types of PA that are at the base of the recommendations for PA in the Hindustani-Surinamese and African-Surinamese populations living in the Netherlands. Marked differences were observed between these populations for the various domains. In addition, ethnic differences were observed regarding frequency, intensity and duration within the domains. Hindustani-Surinamese women reported lower levels of PA in all domains (with the exception of occupational PA), although the magnitude of the differences varied between the considered domains and activities. Lower levels of recommended PA for the other Surinamese groups were also observed although these were not statistically significant.

The domain of commuting PA was found to be popular in the Dutch participants, therefore contributing to the ethnic differences. In contrast, the longer duration of vigorous occupational PA in the Surinamese groups did mitigate ethnic differences in recommended PA to some extent. Finally, culturally-specific types of physical activities, such as yoga and dancing, also mitigated ethnic differences in recommended PA, although this was only slight. The ethnic differences were not accounted for by differences in level of education.

### Discussion of key findings

#### Recommended physical activity

The levels of recommended PA in the Dutch participants of this study are similar to levels found in previous reports [20,21]. The ethnic differences observed between Hindustani-Surinamese and Dutch women and the lack of significant differences between the African-Surinamese and Dutch women are comparable to the differences in PA observed between groups of similar origin in the UK [6,22]. Among others, the Health Survey for England (HSE) found that women of South Asian descent were less active compared with their white British counterparts and with the general UK population [5]. This suggests that women of South Asian descent, such as the Hindustani-Surinamese women in the Netherlands may need extra attention regarding PA stimulation and intervention.

Furthermore, the overall levels of recommended PA were also found to be lower in the African-Surinamese population when compared with the Dutch. This is in contrast with the HSE findings for both men and women of African-Caribbean descent; the HSE data from 2004 found that African-Caribbean people had slightly higher levels of recommended PA compared with the general population.

### Contribution of domains and activities on recommended physical activity

The effect of some individual PA domains, such as active commuting and leisure time to achieve recommended levels of PA has been previously described [23-25]. For example, in a large Californian telephone survey, Berrigan *et al*. showed that the added question about non leisure time walking and cycling (e.g. active transportation) was responsible for reducing ethnic differences in the adherence to the PA recommendation [23]. Our study also shows how much the effect of PA in multiple domains - in relation to the recommended level of PA in a multiethnic population - can contribute. Given the large variations we found between domains, our results suggest that a valid estimation of the total level of PA requires the inclusion of a broad range of domains in order to measure PA.

Some authors recommend culturally adapting the PA questionnaire by adding new or additional activities or by replacing population-specific activities with similar intensity substitute activities [26,27]. In our study, we added the questions on specific activities for all participants without the substitution of similar intensity activities for the various ethnic groups and studied the simultaneous effect of these culturally-specific activities on the recommendation. The results confirm the importance of making cultural adaptations in PA questionnaires.

Some limitations of this study do need to be mentioned. Firstly, this study was based on self-reported levels of PA, which may be subject to response bias - due both to recall bias and social desirability [28]. Respondents may therefore have been more inclined to report their desired level of PA or highest level of PA instead of the actual level they performed. In addition, previous studies have shown that the amount of bias resulting from social desirability may differ between population groups; although we suspect this bias to be small, some studies found that ethnic minorities tend to answer in a more socially desirable way [29,30]. This then implies that the reported levels of PA and recommended PA may have been overestimated to a larger extent in the ethnic minority groups compared to the ethnic Dutch group. However, we do not think that this has affected the main conclusions regarding the variations between domains and specific activities.

Secondly, we added two culturally-specific activities (yoga and dancing) that are known to be popular in the Surinamese population. While there is a risk of missing other culturally-specific physical activities, we did include an open-ended question, which did not identify any other major culturally-specific PA. This finding suggests that yoga and dancing cover most of the cultural differences in leisure time PA in this population group.

Thirdly, our study focussed on specific ethnic minority groups living in the Netherlands. The exact patterns of PA we identified in the general population and in the ethnic groups could possibly be different in other population groups or in other locations. Further research, similar to this study, is necessary in order to identify patterns in PA among ethnic groups who are either not living in their native country or living under other circumstances.

## Conclusion and recommendations

Our results suggest that estimates of the size or extent of ethnic differences in the prevalence of recommended PA are dependent on the domains and activities included in the relevant questionnaire. Consequently, studies on differences in PA levels between ethnic groups should include multiple domains of PA. In particular, including the domain of occupational PA appears to be important here, as well as including household PA and culturally-specific activities like yoga and dancing in ethnic minority populations.

A comprehensive description of the different domains and activities would provide a broader evidence base so that tailored PA interventions for specific ethnic groups could be made.

## Competing interests

The authors declare that they have no competing interests.

## Authors' contributions

JM carried out the statistical analyses and drafted the manuscript. All authors (JM, IV, CA, AK, and KS) participated in interpretation of the data and contributed critical revision for intellectual content of the manuscript. All authors read and approved the final manuscript.
